# Digital Demodulation Method and Application of a PWM-Excited Differential Self-Inductive Displacement Transducer

**DOI:** 10.3390/s26092751

**Published:** 2026-04-29

**Authors:** Hui Guo, Boqiang Shi, Hu Chen, Bingbing Liu

**Affiliations:** School of Mechanical Engineering, University of Science and Technology Beijing, Beijing 100083, China; d202410315@xs.ustb.edu.cn (H.G.);

**Keywords:** differential self-inductive displacement transducer, symmetric complementary square-wave signal, digital demodulation, microcontroller-based measurement, displacement measurement

## Abstract

Accurate measurement of spool displacement is essential for achieving high-performance closed-loop control and condition monitoring in hydraulic systems. However, conventional inductive displacement transducers typically rely on sinusoidal excitation and complex analog signal conditioning circuits, resulting in higher hardware cost and limited system integration. To address these issues, this paper proposes a software-based demodulation method for a differential inductive displacement transducer under symmetric complementary square-wave excitation. First, the structure and operating principle of the transducer are analyzed, and an electromagnetic model describing the nonlinear relationship between coil inductance and the position of the inductive core is established, along with its electrical characteristics. Then, a simplified signal acquisition circuit is designed to enable digital extraction of inductance variations using a microprocessor. Compared with conventional approaches, the proposed scheme significantly reduces hardware complexity and cost while being more suitable for embedded system integration. A simulation model is developed to analyze the inductance variation and to validate the proposed hardware circuit. In addition, a test platform is built to conduct static calibration and dynamic response experiments. The experimental results show that the proposed method achieves a linearity of 2.36% and a sensitivity of 155.6 mV/mm and exhibits strong robustness against switching noise. Finally, application tests in a hydraulic valve system demonstrate that the proposed transducer and demodulation method enable accurate and stable spool position measurement, providing a low-cost and easily integrated solution for embedded hydraulic control systems.

## 1. Introduction

Inductive displacement transducers are non-contact measurement devices characterized by minimal temperature sensitivity, high reliability, exceptional sensitivity, excellent linearity, low cost, and extended service life [1,2]. They are particularly suitable for operation in harsh environments with severe temperature fluctuations, oil contamination, and mechanical vibrations [3,4]. Inductive displacement transducers can be categorized into self-inductive and mutual-inductive types, though both operate on the principle of measuring position through changes in coil inductance caused by variations in the position of the inductive core. Owing to these characteristics, inductive displacement transducers have become a mainstream solution for spool position measurement in hydraulic valves and serve as key components for achieving high-precision closed-loop control in hydraulic systems [5,6].

To further optimize the performance of inductive transducers, extensive research has been carried out by scholars both domestically and internationally, with particular emphasis on the evolution of structural topology and magnetic materials. In theoretical modeling, Ren improved the theoretical model of the self-inductive transducer by incorporating the effects of eddy currents in the magnetic core [7]. In terms of materials science, Mirzaei et al. achieved high-precision temperature compensation by optimizing the material properties of the inductive core [8]. In terms of structural design, researchers have improved sensitivity and accuracy by modifying the end configuration of the core [9,10] or incorporating magnetic field shielding structures [11,12]. However, conventional linear variable differential transformers (LVDTs) suffer from an inherent limitation: the ratio between the physical size of the transducer and its linear measurement range (i.e., the stroke ratio) is relatively large [13]. Consequently, in applications requiring large measurement strokes, the transducer size increases substantially, making it difficult to meet the stringent requirements of modern industrial actuators for compactness and embedded integration.

The measurement accuracy of inductive displacement transducers depends not only on their physical structure but also critically on the performance of the signal conditioning circuitry. Ren et al. investigated a signal processing circuit using a three-coil inductive displacement transducer as the subject of their research, demonstrating that this circuit effectively enhances the transducer’s performance [14]. Existing studies have primarily focused on improving demodulation techniques, such as timer-based AM demodulators [15] and AC excitation signal generation circuits [16]. To achieve higher system integration, several approaches utilize dedicated integrated circuits (e.g., AD598 or AD630) for signal conditioning and demodulation [17,18]. However, this technical route faces a dual challenge in terms of cost control and reliability. The adoption of high-performance integrated chips significantly increases the hardware cost of individual transducers, whereas low-cost solutions based on discrete components often suffer from limitations such as pronounced temperature drift and poor immunity to electromagnetic interference [19]. Although previous studies have investigated the influence of excitation frequency on sensitivity [20,21], achieving substantial circuit simplification through simplified circuit topology remains a critical challenge that has yet to be fully addressed.

With the deep integration of Industry 4.0 and intelligent hydraulic technologies, displacement sensors are evolving toward intelligent, miniaturized, and highly integrated architectures [22]. This trend not only requires these devices to achieve a higher stroke-to-length ratio but also places stricter constraints on the spatial footprint and integration level of the associated control circuitry. The authors of [23] proposed a differential variable reluctance transducer (DVRT), which is derived from a conventional LVDT by removing the central winding and retaining only the two end windings. Flammini et al. further demonstrated that eliminating one winding in the LVDT structure allows the DVRT to achieve a larger stroke-to-length ratio [3]. In addition, the central tap configuration provides enhanced immunity to common-mode disturbances, such as environmental temperature fluctuations [24]. However, most existing DVRT implementations still rely on complex phase-sensitive demodulation circuits, which significantly hinder the realization of embedded and highly integrated sensor–controller architectures [25].

To address the above issues, this paper proposes a PWM-compatible digital demodulation method suitable for differential self-inductance displacement transducers. The main contributions are summarized as follows:

(1) A simplified signal acquisition circuit based on a differential self-inductance transducer is developed, capable of directly interfacing with a microprocessor;
(2) A software-based demodulation method suitable for symmetric complementary square-wave signal is proposed, eliminating the need for traditional analoge demodulation circuits;
(3) The proposed method is validated through simulation and experimental studies, and its effectiveness is further demonstrated in a closed-loop control system for hydraulic valves.

The structure of this study is organized as follows: Section 2 outlines the physical structure and operational principles of the transducer; Section 3 presents the mathematical derivation of electromagnetic characteristics and detailed design of the measurement circuit; Section 4 validates the feasibility of the processing circuit and algorithm through a simulation model; Section 5 constructs an experimental platform based on the S32K microcontroller, verifying the method’s effectiveness by comparing simulation and experimental data; and Section 6 integrates the developed sensor into a hydraulic valve to conduct position closed-loop control experiments, thereby validating the system’s comprehensive performance in practical engineering applications.

## 2. Structure and Operating Principle of a Solenoid Differential Self-Inductive Displacement Transducer

### 2.1. Structure of Displacement Transducer

**Figure 1:**
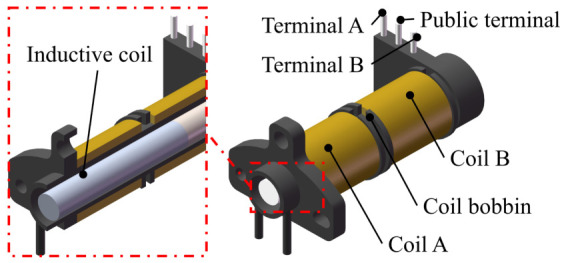
Structure of a differential self-inductive displacement transducer.

As shown in Figure 1, the differential self-inductive displacement transducer adopted in this work shares the same basic configuration as a DVRT (differential variable reluctance transducer) and mainly consists of three components: a coil bobbin, a pair of symmetrically connected series coils, and an inductive core. The bobbin serves as the structural support for coil winding and circuit integration. It is fabricated from a high-performance engineering plastic. The selected material exhibits excellent non-magnetic characteristics, effectively preventing disturbances to the sensing magnetic field. The bobbin is equipped with three copper terminals; the upper ends are soldered to the coils, while the lower ends are directly soldered onto the system PCB. This configuration ensures robust mechanical fixation and reliable integration with the signal conditioning circuitry. Two identical coils, wound with enameled copper wire, are symmetrically arranged in the two chambers inside the bobbin. This symmetrical structure is essential for achieving differential sensing and suppressing common-mode interference. The movable core is fabricated from silicon steel with high saturation magnetic flux density and excellent magnetic permeability. During assembly, precise positioning ensures coaxial alignment between the sensing core and the coil bobbin. Consequently, the axial displacement of the core within the bobbin directly changes the self-inductance of the two coils.

### 2.2. Working Principle of Displacement Transducer

**Figure 2:**
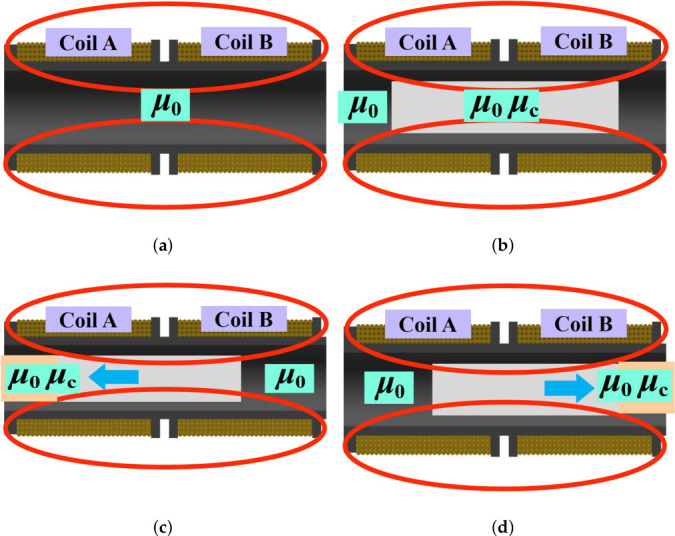
Operating principle of a differential self-inductive displacement transducer. (**a**) Hollow solenoid. (**b**) Core moves to the middle. (**c**) Core moves to the left. (**d**) Core moves to the right. In the figure, the red circles represent magnetic flux.

Under AC excitation, alternating magnetic fields are generated around the two coils of the differential self-inductive transducer, as shown in Figure 2a. The axial movement of the inductive core alters the magnetic permeability within each coil, thereby causing corresponding changes in their inductance.

Assuming that the magnetic field inside the solenoid is uniform, the inductance of a hollow solenoid can be determined by the following expression [26]:

(1) $$L=\frac{\Psi }{I}=\frac{N\Phi }{I}=\frac{NBS}{I}=\frac{\mu_{0}N^{2}S}{l}$$

where *L* is the coil inductance; *N* and *l* are the number of turns and the coil length, respectively; Ψ is the equivalent magnetomotive force; Φ is the magnetic flux through the coil; *B* denotes the axial magnetic flux density; *S* is the coil’s cross-sectional area; and $\mu_{0}$ is the permeability of free space.

For the displacement sensor investigated in this study, variations in the position of the sensing core modify the effective magnetic permeability, which in turn leads to corresponding changes in the inductance of the coils, as described by Equation (1). As illustrated in Figure 2b, when the inductive core is located at the midpoint between the two coils, the effective magnetic permeabilities associated with coils A and B are identical, resulting in identical inductance. When the core moves to the left, the permeability associated with coil A increases while that of coil B decreases, resulting in increased inductance in coil A and decreased inductance in coil B, as shown in Figure 2c. Conversely, when the core moves to the right, the opposite trend occurs, as illustrated in Figure 2d. In the subsequent signal conditioning stage, these inductance variations are converted into voltage signals suitable for direct microprocessor processing, enabling accurate displacement measurement.

## 3. Electromagnetic Characteristics and Signal Acquisition

### 3.1. Electromagnetic Characteristics

**Figure 3:**
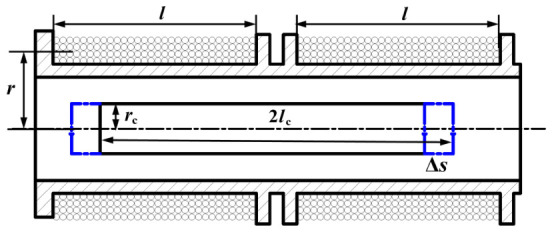
Structure parameters of the coil. In the figure, the blue line represents the left and right displacement of the inductive core.

In the integrated application of the solenoid differential self-inductive transducer, the inductive core is rigidly connected to the hydraulic valve spool through a non-magnetic connecting rod. To ensure measurement accuracy and minimize disturbances caused by lateral forces, coaxial alignment must be maintained among the coil bobbin, inductive core, connecting rod, and the motion axis of the valve spool. Since the focus of this study is the displacement sensor, only a schematic diagram of the transducer is presented for clarity, as shown in Figure 3. Each coil has a length of *l* and a number of turns *N*. The coil winding radius is denoted by *r*, the radius of the sensing core by $r_{c}$, and the magnetic permeability of the sensing core by $\mu_{c}$.

Assuming that the displacement of the inductive core is Δs, the inductance of coil A can be approximately derived from Equation (1) and [21] as follows:

(2) $$L_{A}=\frac{\mu_{0}N^{2}\pi }{l^{2}}\left[lr^{2}+\mu_{c}r_{c}^{2}\left(l_{c}+\Delta s\right)\right]$$

Similarly, the inductance of coil B is

(3) $$L_{B}=\frac{\mu_{0}N^{2}\pi }{l^{2}}\left[lr^{2}+\mu_{c}r_{c}^{2}\left(l_{c}-\Delta s\right)\right]$$

The variations in inductance of the two coils are given by

(4) $$\Delta L=L_{A}-L_{B}=2\frac{\mu_{0}\mu_{c}N^{2}\pi r_{c}^{2}}{l^{2}}\Delta s$$

As indicated by Equation (4), the inductance variation ΔL is proportional to the displacement change Δs. Therefore, the inductance sensitivity can be expressed as follows:

(5) $$\Delta K=\frac{\Delta L}{\Delta s}=2\frac{\mu_{0}\mu_{c}N^{2}\pi r_{c}^{2}}{l^{2}}$$

Equation (5) indicates that the inductance sensitivity depends on the relative permeability of the sensing core, the core radius, and the number of turns.

### 3.2. Electrical Characteristics

**Figure 4:**
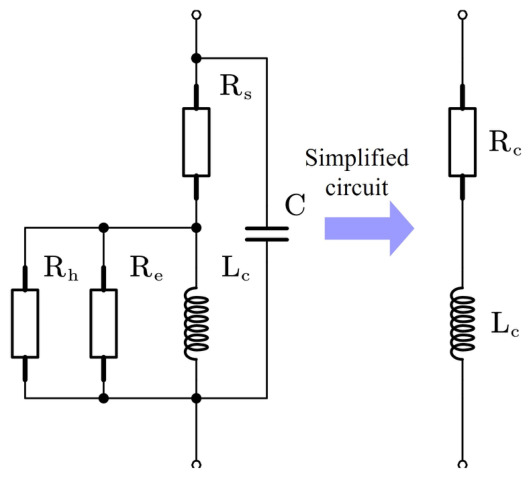
Simplified model of the inductance coil.

The coils in the solenoid differential self-inductive displacement transducer are not ideal inductive elements. In practice, the equivalent electrical model of the coil includes the copper loss resistance $R_{c}$, the eddy current loss resistance $R_{e}$, the hysteresis loss resistance $R_{h}$, and the inter-turn capacitance and the distributed capacitance of the connecting cables *C*. All loss resistances in the coil and the inductive core are equivalently represented by a single resistance $R_{s}$. Although the parallel parasitic capacitance *C* may affect the sensor sensitivity, it remains constant and does not vary with displacement in the proposed design. After sensitivity calibration, its influence can therefore be neglected in the analysis. Consequently, the equivalent circuit of the inductive coil can be simplified as shown in Figure 4 [27]. As this topic has been extensively studied, it is not discussed further in this paper.

### 3.3. Signal Acquisition Circuit Design

**Figure 5:**
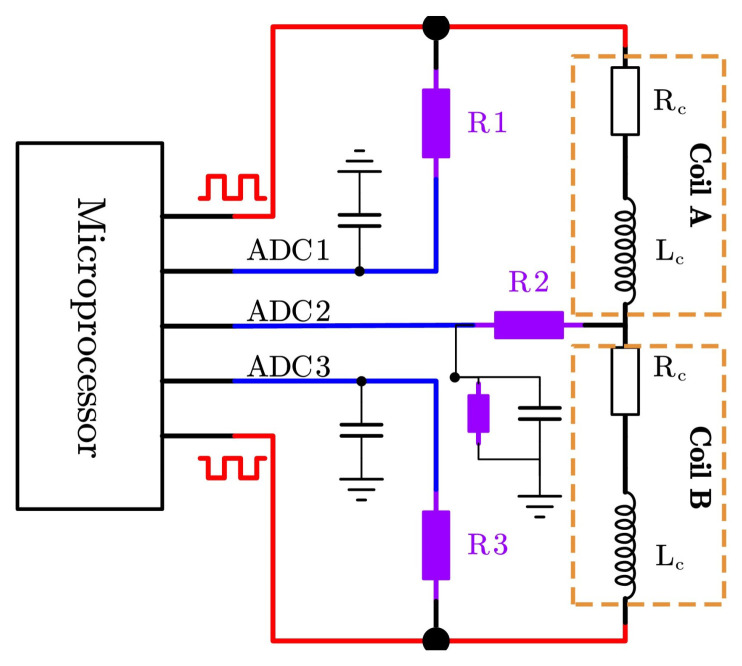
Signal acquisition circuit.

To overcome the limitations of traditional modulation–demodulation circuits, including complex hardware, high power consumption, and limited integration capability, a signal processing circuit is proposed, as shown in Figure 5. Here, $R_{1}$, $R_{2}$, and $R_{3}$ denote the auxiliary resistors in the signal-conditioning network. The proposed scheme is based on the direct electrical interface between the displacement transducer and the microprocessor, combined with a simple filtering circuit. Two complementary PWM signals generated by the microprocessor via two I/O pins are used as the excitation source, with a fixed duty cycle of 50% and a frequency of 10 kHz. These parameters were selected as a practical engineering compromise by considering measurement refresh rate, timing implementation, ADC (analog-to-digital converter) synchronization, and signal symmetry rather than through a dedicated optimization study. The voltages at the two terminals and the common terminal of the sensor are sampled in real time by the ADC channels (ADC1, ADC2, and ADC3), respectively. By analyzing the relationship between the common-terminal voltage and the terminal voltages, the displacement related to the inductance difference between the two coils can be accurately obtained.

To overcome the limitations of traditional modulation–demodulation circuits, such as complex hardware implementation, high power consumption, and limited integration capability, a signal processing circuit is proposed, as shown in Figure 5. Here, $R_{1}$, $R_{2}$, and $R_{3}$ denote the auxiliary resistors in the signal-conditioning network; $V_{\mathrm{in}}$ is the differential symmetric complementary square-wave signal voltage provided by the microprocessor; and $V_{A}$ is the voltage at node A, namely the intermediate sensing node used for subsequent signal analysis.

Over one period of the PWM signal, it is assumed that during the first half of the period, a high-level voltage is applied to coil A, while coil B receives a low-level voltage [28,29]. According to Kirchhoff’s voltage law (KVL), the equation can be written as

(6) $$V_{in}=2iR_{c}+2L_{c}\frac{di}{dt}$$

where $V_{\mathrm{in}}$ is the differential PWM excitation voltage provided by the microprocessor. $R_{c}$ denotes the equivalent resistance, and $L_{c}$ denotes the inductance of each coil. During the first half of the period T/2, assuming that the inductive core moves to the right, the voltage at the common terminal of the two coils, $V_{A}$, is given by

(7) $$V_{A}=iR_{c}+\left(L_{c}-\Delta L\right)\frac{di}{dt}$$

where ΔL denotes the inductance change of a single coil. By combining Equations (6) and (7), it follows that

(8) $$V_{A}=\frac{V_{in}}{2}-\Delta L\frac{di}{dt}$$

When the PWM signal switches between high and low levels, the circuit can be considered to be in a steady state according to Equation (6). Therefore, with the initial condition i(0)=V/2R, the solution of Equation (6) can be written as

(9) $$i=\frac{V_{in}}{2R_{c}}\left(1-2e^{-\frac{R_{c}}{L_{c}}t}\right)$$

Equation (8) can be rewritten as

(10) $$V_{A}=\frac{V_{in}}{2}+\Delta L\frac{V_{in}}{L_{c}}e^{-\frac{R_{c}}{L_{c}}t}$$

Similarly, in the second half of the cycle T/2, the voltage at the common terminal $V_{B}$ can be expressed as

(11) $$V_{B}=\frac{V_{in}}{2}-\Delta L\frac{V_{in}}{L_{c}}e^{-\frac{R_{c}}{L_{c}}t^{\prime }}$$

Since the duty cycle of the PWM signal is 50%, $t=t^{\prime }=T_{h}$ can be assumed. The voltages $V_{A}$ and $V_{B}$ are sampled by the ADC module of the microprocessor. Therefore, the amplitude of the common-terminal voltage within one PWM period can be expressed as

(12) $$\Delta V=V_{A}-V_{B}=2\Delta L\frac{V_{in}}{L_{c}}e^{-\frac{R_{c}}{L_{c}}T_{h}}\approx K\Delta L$$

As indicated by Equations (10) and (11), when the inductive core moves to the right, the common-terminal voltage exhibits a high voltage level during the first half-cycle and a low voltage level during the second half-cycle. The phase of this voltage is consistent with that of the ADC1 signal and opposite to that of the ADC2 signal. When the inductive core moves to the left, the phase relationship is reversed. Furthermore, the voltage amplitude is proportional to the inductance variation. Therefore, the displacement magnitude can be determined from the voltage amplitude of ADC2 within one PWM period, while the displacement direction can be identified from the phase relationship between the ADC1 and ADC2 signals.

### 3.4. Signal Acquisition Software Design

**Figure 6:**
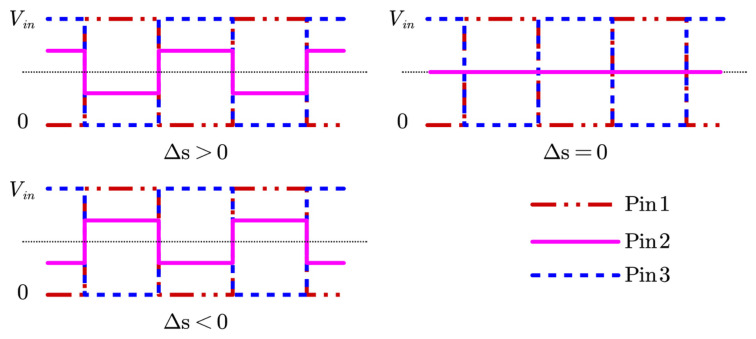
Schematic of transducer-acquired voltage signals.

The previous section covered hardware circuit design and the corresponding mathematical derivations. This section focuses on software-based signal demodulation. Based on the analysis presented earlier, the raw signal acquired by the microprocessor can be simplified to the form shown in Figure 6. Taking into account the characteristics of this signal, this paper proposes a software-based demodulation method for the S32K1xx series of microprocessors.

**Figure 7:**
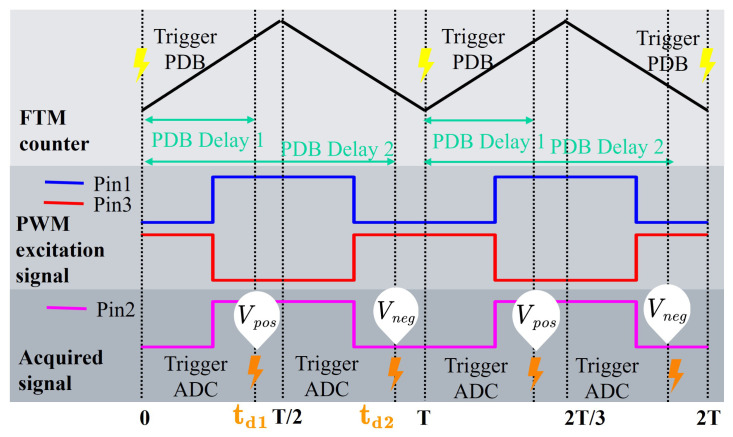
Signal demodulation timing diagram.

This method requires coordinated operation of the FTM (FlexTimer module), PDB (programmable delay block), and ADC (analog-to-digital converter) modules within the MCU (microcontroller unit). By utilizing precise timing alignment, displacement signal demodulation without delay, and automatic direction identification are achieved. The timing principle is illustrated in Figure 7. When the FTM counter reaches the beginning of its period (i.e., t=0), a hardware synchronization pulse is generated to trigger the PDB counter. The PDB is configured in dual-trigger mode, with two delay parameters $t_{d1}$ and $t_{d2}$. When the delay times are reached, the ADC sampling function is triggered, enabling instantaneous sampling during the steady-state intervals of the high-level and low-level excitation signals, respectively. The purpose of setting two delay times is to ensure that the sampling points are always located within the flat-top region of the signal envelope, thereby suppressing high-frequency noise at the physical level.

Let $V_{\mathrm{pos}}$ denote the sampled value during the positive half-cycle of the excitation signal, and $V_{\mathrm{neg}}$ denote the sampled value during the negative half-cycle. The displacement demodulation result $L_{\mathrm{raw}}$ can be expressed as

(13) $$L_{\mathrm{raw}}=V_{\mathrm{pos}}-V_{\mathrm{neg}}$$

Under this algorithm, the absolute value represents the deviation of the spool from its neutral position, while the algebraic sign inherently contains phase information, enabling automatic determination of displacement direction. To further improve signal quality, a first-order IIR low-pass filter is applied to the demodulated signal. Its discrete form is given by

(14) $$y\left(n\right)=\alpha \cdot L_{\mathrm{raw}}\left(n\right)+\left(1-\alpha \right)\cdot y\left(n-1\right)$$

where α is the filter coefficient. This approach eliminates the need for conventional diode rectification and RC filtering circuits, thereby avoiding the phase lag introduced by analog demodulation.

## 4. Simulation Analysis

### 4.1. Development of the Simulation Model

**Figure 8:**
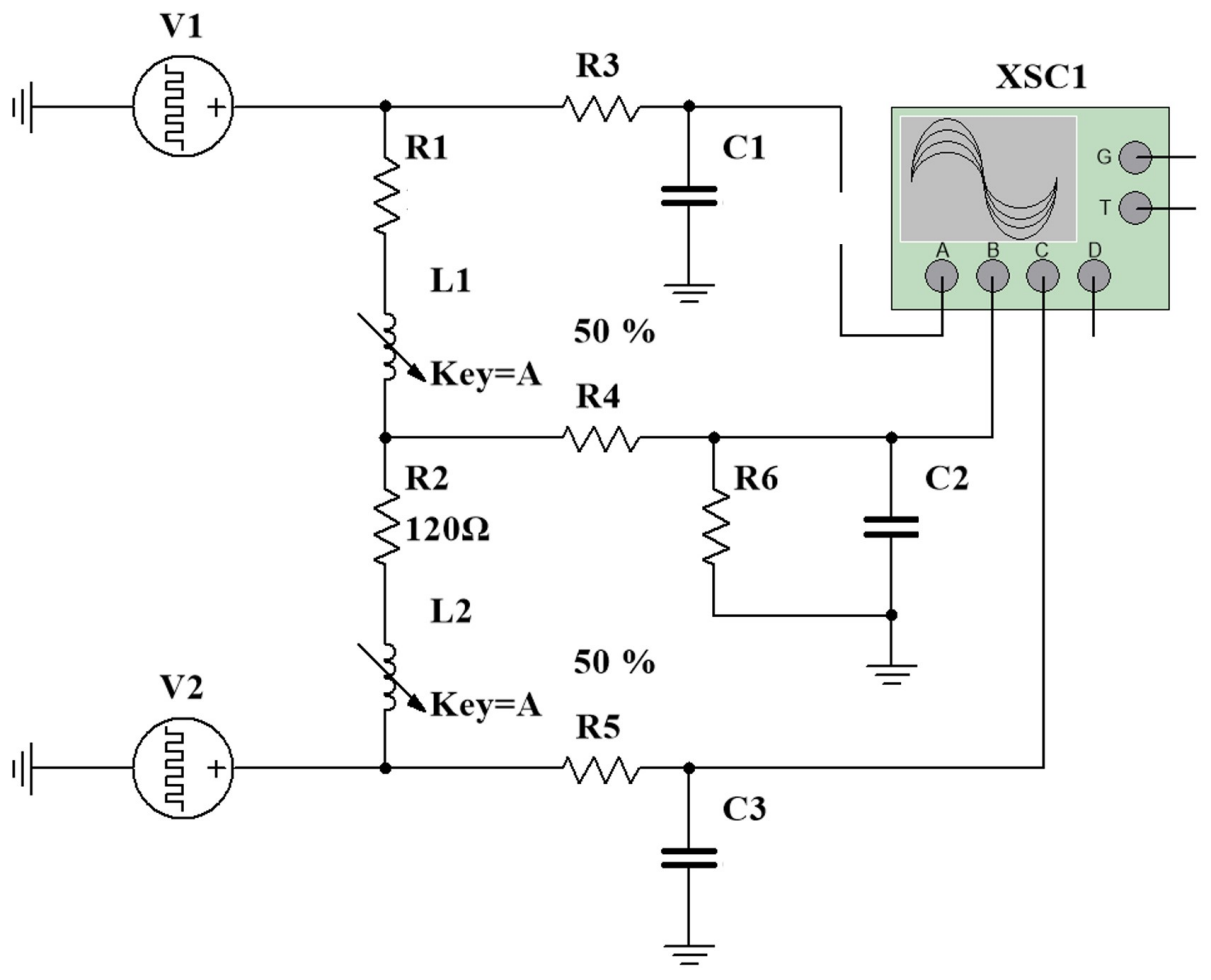
Simulation model of the signal acquisition circuit.

To verify the above measurement principle, a signal acquisition circuit model was established in the Multisim simulation platform, as shown in Figure 8. In the simulation model, two complementary square-wave sources, $V_{1}$ and $V_{2}$, were used to emulate the symmetric complementary square-wave signals generated by the microprocessor. The dual-coil structure of the inductive displacement transducer was represented by equivalent resistances $R_{1}$ and $R_{2}$ together with variable inductances $L_{1}$ and $L_{2}$, which simulate the differential inductance variations caused by the displacement of the sensing core. RC filter networks composed of $R_{3}C_{1}$, $R_{5}C_{3}$, and $R_{4}C_{2}$ were connected at the signal output terminals to suppress high-frequency carrier components. Finally, the voltage waveforms at the simulated ADC sampling nodes were monitored in real time using the four-channel oscilloscope (XSC1).

**Figure 9:**
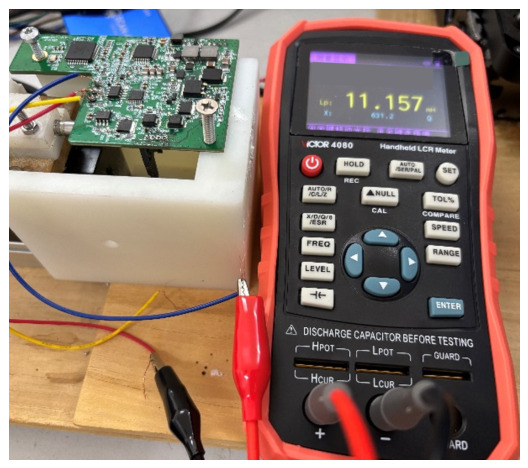
Testing of the inductance of VICTOR4082.

**Figure 10:**
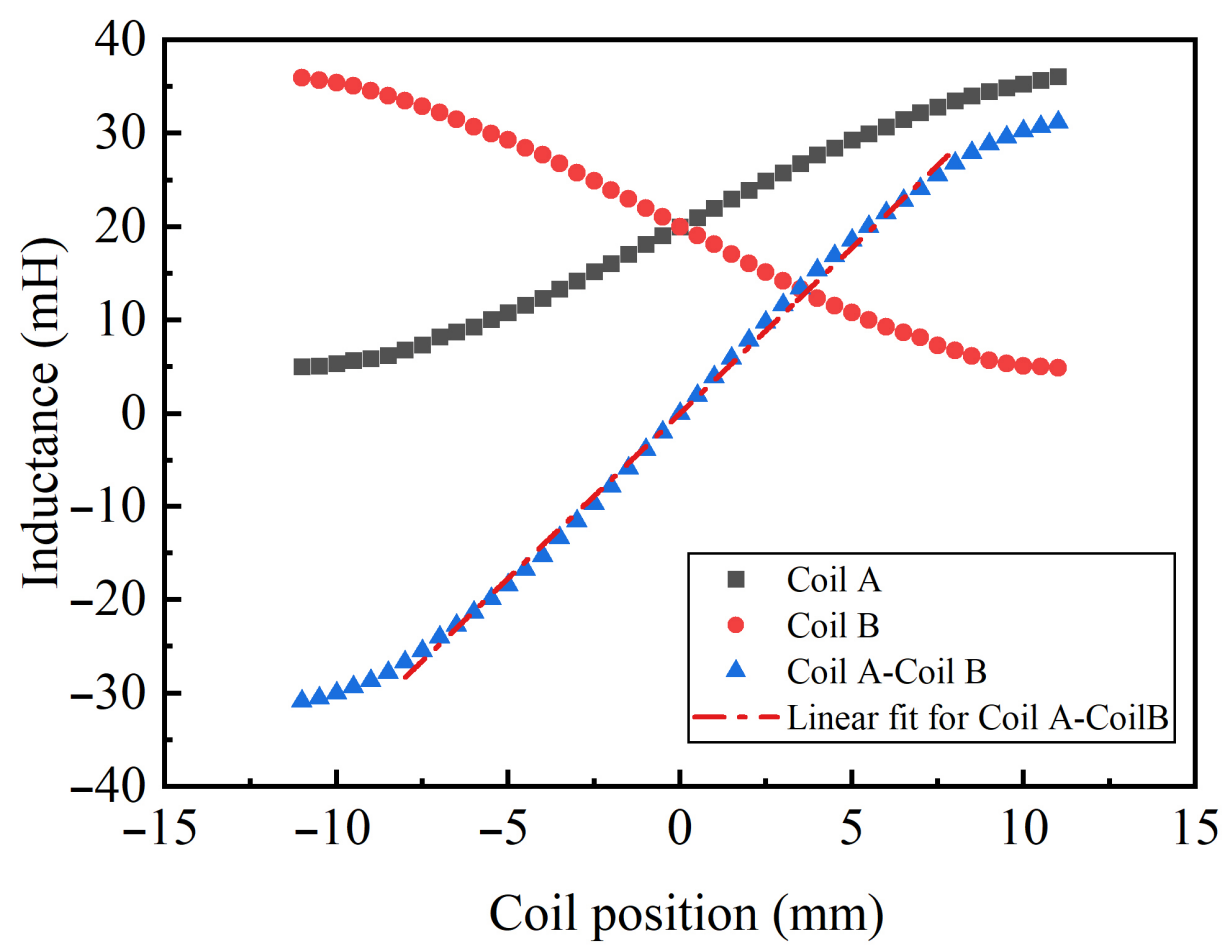
The relationship between coil position and inductance in VICTOR4082.

Due to the difficulty in directly establishing the coupling relationship between the mechanical displacement of the inductive core and the coil inductance in the simulation model, a precision LCR meter (inductance–capacitance–resistance meter, VICTOR 4082), as shown in Figure 9, was employed to experimentally measure the inductances of the two coils and their differential inductance at different positions. To reduce experimental errors, the measurements were repeated three times under identical test conditions, and the average values were used as the final results. The experimental results are presented in Figure 10. As the position of the sensing core varies, the differential inductance exhibits a high degree of linearity over a displacement range of ±8 mm. The fitted relationship within this range can be expressed as

(15) ΔL=3.541x+0.009,

where ΔL is the differential inductance, *x* is the displacement of the sensing core. This result is generally consistent with the variation trend reflected by Equation (5). However, due to practical factors such as the actual coil geometry, fringe-field effects, and manufacturing and assembly errors, a rigorous theoretical numerical calculation and quantitative comparison were not further carried out in this study. The fitting results show that the coefficient of determination $R^{2}$ is 0.998, indicating a high degree of agreement between the experimental data and the linear model and demonstrating that the proposed transducer exhibits good linearity within the displacement range of ±8 mm. These measured data provide accurate parameters for the subsequent circuit simulations, thereby ensuring the reliability of the simulation results.

### 4.2. Simulation Results Analysis

**Figure 11:**
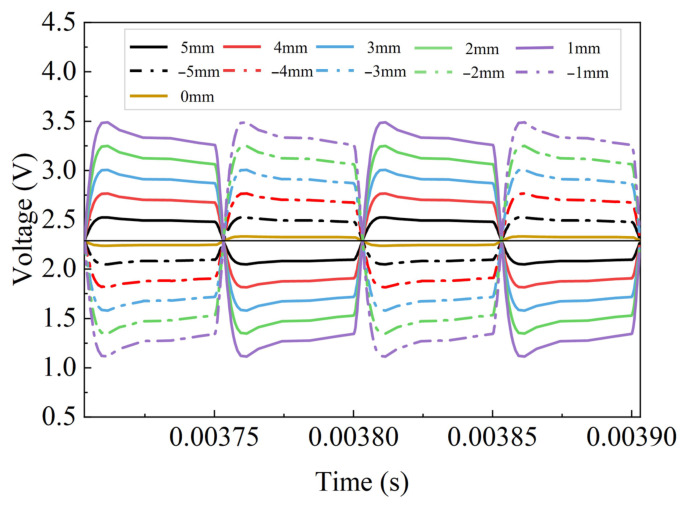
Output voltage at different positions (simulation results).

Based on the established simulation model, the position of the inductive core is characterized by varying the inductance parameters $L_{1}$ and $L_{2}$. Figure 11 presents the voltage distribution of the inductive sensing circuit at different core positions. It can be observed from the figure that the voltage is not constant within either the positive or negative half-cycle. However, at the same position, the voltage values remain consistent from cycle to cycle, and the voltage variation trends within the positive and negative half-cycles are also consistent. The scheme described in Section 3.4 uses a precise delay trigger in the microprocessor to sample the voltage at a fixed instant within the positive and negative half-cycles of each cycle, thereby ensuring that the sampled voltage remains consistent at the same position. In addition, it can be observed that when the inductive core is located at the neutral position, the output signal of ADC2 remains constant within each cycle, indicating that the differential inductive structure is in a balanced state. Taking the neutral position as the reference, the amplitude of the ADC2 output signal increases monotonically with displacement as the core moves to the left, reaching a maximum value of 3.523 V at a certain position. When the core moves to the right, the system exhibits a symmetric behavior, where the signal amplitude also increases with displacement and reaches the same maximum value. This symmetry indicates that the amplitude of the ADC2 signal only reflects positional information. Due to the limitations of the Multisim platform, the simulation model cannot directly provide the quantitative relationship between the sensor position and the output voltage.

Nevertheless, the simulation results are sufficient to verify the feasibility of the proposed hardware circuit. In the following section, experimental validation will be conducted based on the S32K144 microcontroller.

## 5. Experimental Validation

### 5.1. Establish the Test Platform

**Figure 12:**
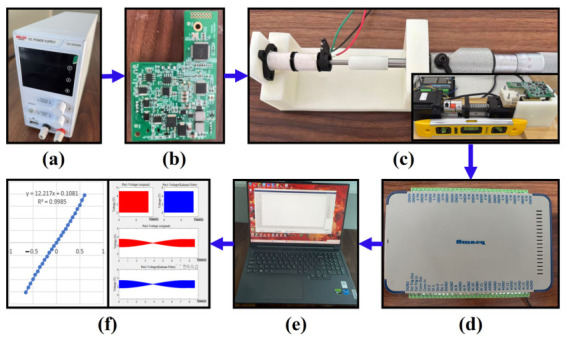
Experimental test flowchart. (**a**) Power. (**b**) A PCB with the designed circuit. (**c**) Testing platform. (**d**) Data acquisition Instrument. (**e**) Host computer. (**f**) Collection and calibration results.

The agreement between the theoretical analysis and simulation results provides strong evidence for the validity of the proposed signal processing circuit for the solenoid-based differential inductive sensor. Based on the proposed circuit design, a PCB controller integrating the signal processing circuit was implemented, and a dedicated experimental platform for displacement measurement was established. The experimental system incorporates two displacement actuation mechanisms. Specifically, a stepper-motor-driven linear guide system is employed for dynamic stroke testing, while a manual micrometer displacement device is utilized for high-precision static calibration. The overall experimental setup is illustrated in Figure 12.

In the experimental system, a regulated power supply is used to power the PCB controller. The onboard microcontroller generates the excitation signals for the sensor and configures the input/output pins according to the previously designed software-based demodulation scheme. The sensor voltage signals are acquired using a data acquisition (DAQ) system (smacq-USB-5321) with a sampling rate of 200 kSa/s/ch, a 12-bit resolution, and an input range of ±10 V/±5 V and are subsequently processed by the host computer to reconstruct the corresponding displacement.

### 5.2. Dynamic Travel Test

**Figure 13:**
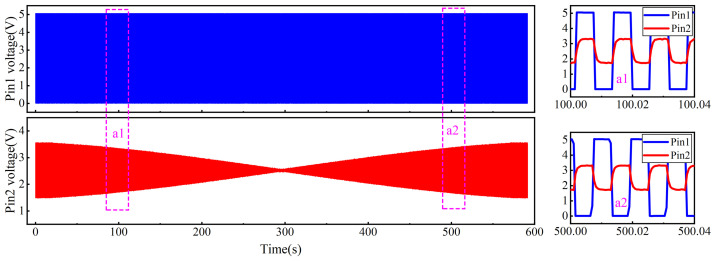
Pin raw voltage signal.

To evaluate the dynamic performance of the transducer, the inductive core was fixed to the moving platform of a linear guide. A stepper motor was used to drive the inductive core at a constant speed of 45mm/s, moving from right to left across the full measurement range of the displacement transducer. Meanwhile, the voltages at ADC1 and ADC2 were synchronously acquired using a data acquisition instrument. According to the Nyquist sampling theorem, the sampling frequency should be at least twice the highest frequency component of the signal. To more accurately capture the characteristics of the PWM signal, the sampling frequency was set to ten times the highest signal frequency. The acquired raw signals are shown in Figure 13.

The experimental voltage signals exhibited close agreement with those predicted by the simulation model. When the inductive core was positioned at the geometric center of the electromagnet, the voltage amplitude at Pin 2 over a single cycle was approximately 0.02 V. As the core gradually displaced to the left or right, the voltage amplitude increased roughly linearly. Near the extremities of the stroke, the amplitude exhibited non-linear growth and eventually saturated, reaching a maximum of approximately 7.6 V. In contrast, the voltage at Pin 1 remained essentially constant across the full range of motion. A magnified view of the measured data reveals that when the core is symmetrically positioned on either side of the electromagnet’s center, the voltage amplitude at Pin 2 is identical, while the phase relative to the voltage at Pin 1 differs by $180^{\circ }$.

**Figure 14:**
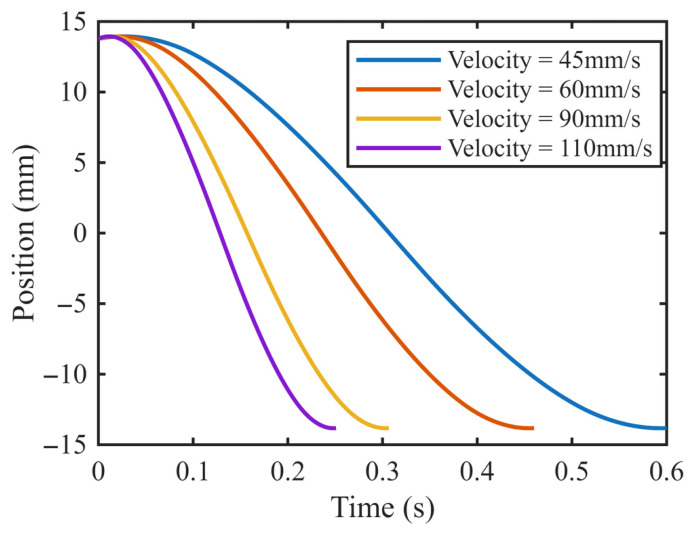
Processed displacement transducer voltage signals at various spool velocities.

The voltage signals obtained after software demodulation are presented in Figure 14. To assess the dynamic performance of the sensor, the core displacement velocity was varied during testing. Considering that the sensor is intended for hydraulic directional control valves—where the maximum spool velocity is approximately 100 mm/s—the core was moved along the electromagnet’s stroke at velocities of 110 mm/s, 90 mm/s, and 60 mm/s. The corresponding demodulated voltage signals are shown in Figure 14. These results demonstrate that, with the proposed signal conditioning circuit, the displacement sensor meets the performance requirements for accurately measuring the position of a hydraulic valve spool.

### 5.3. Static Calibration

**Figure 15:**
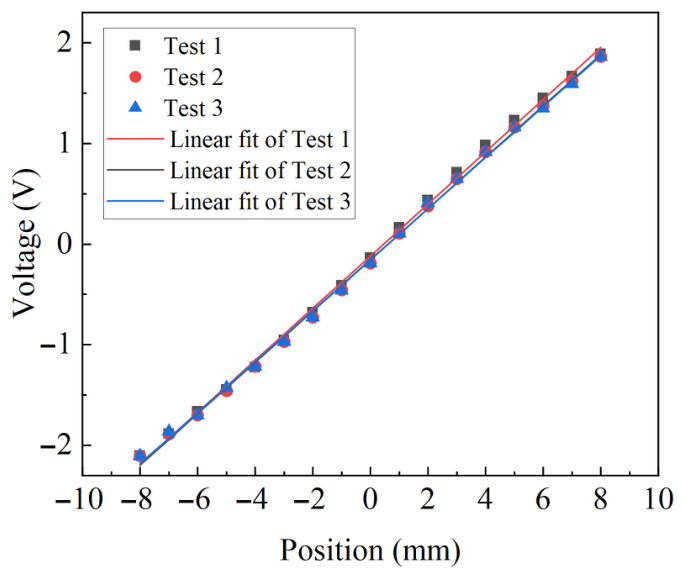
Output voltage at different positions (experimental results).

A static calibration experiment was performed to evaluate the performance of the displacement sensor based on the proposed signal conditioning circuit and processing algorithm. The experimental setup consisted of a solenoid mounting base and a precision micrometer, which was used to accurately control the position of the sensing core. The conditioned voltage signal was transmitted by the microprocessor to the host computer through a serial interface for real-time monitoring and recording. By adjusting the position of the sensing core and measuring the corresponding output voltage, the displacement–voltage characteristic of the sensor was obtained, as illustrated in Figure 15. The obtained data were subjected to linear fitting, and the results are presented in Table 1. All Pearson correlation coefficients, $R^{2}$, and adjusted $R^{2}$ values exceed 0.998, indicating excellent linearity of the proposed sensor.

The linearity of a transducer can be calculated using the following formula:

(16) $$\delta_{L}=\frac{\max\left|y_{i}-y_{fit,i}\right|}{y_{\max}-y_{\min}}\times 100\%$$

The linearity error $\delta_{L}$ is defined as the ratio of the maximum deviation between the measured output and the fitted linear output to the full-scale output. Here, $y_{i}$ denotes the measured output at the *i*-th sampling point; $y_{fit,i}$ represents the corresponding value obtained from the linear fitting model; and $y_{\max}$ and $y_{\min}$ denote the maximum and minimum output values within the measurement range, respectively. According to Equation (16), the linearity error is 2.36%. The sensitivity is defined as the slope of the fitted linear curve, which is 155.6 mV/mm. From the three repeated measurements, the repeatability is good. In summary, the actual response characteristics of the sensor in the proposed measurement system, including its effective measurement range, sensitivity, and linearity, were experimentally validated. These performance indicators satisfy the requirements of hydraulic-valve applications and were further verified in Section 6.

## 6. Engineering Applications

**Figure 16:**
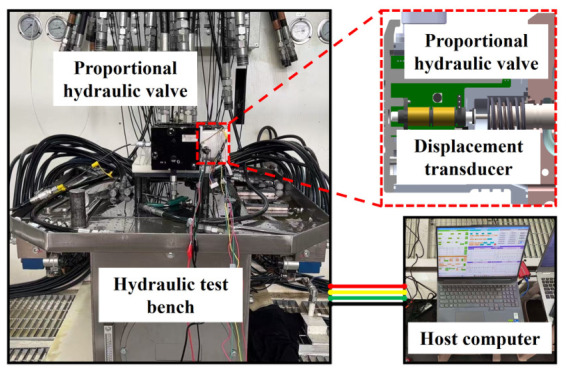
Closed-loop control experiment of spool position in proportional hydraulic valves.

To validate the operational performance of the transducer developed herein and its lightweight signal processing circuit in industrial environments, the system was applied to a closed-loop control experiment for the spool position of a proportional hydraulic valve. The specific experimental system configuration is shown in Figure 16. Mechanically, the transducer’s inductive core is rigidly connected to the main spool of the hydraulic valve through a threaded connection, allowing synchronous axial displacement. The inductive coil bobbin is precisely embedded within the sensing chamber at the end of the valve body. From the hardware perspective, the processing circuit is directly integrated into the valve controller PCB, which is equipped with an S32K microprocessor. This configuration eliminates the need for conventional external signal conditioning modules, thereby achieving a highly integrated system.

In the closed-loop position control system for the hydraulic valve spool, a conventional PID control strategy is employed. The control error e(t) is defined as the difference between the target displacement command and the actual measured displacement. The PID controller then computes the corresponding control action, which is converted into the PWM duty cycle for actuating the solenoid of the proportional hydraulic valve. Based on empirical tuning, the PID parameters are set as follows: proportional gain $K_{p}=20$, integral gain $K_{i}=200$, and derivative gain $K_{d}=0.3$.

**Figure 17:**
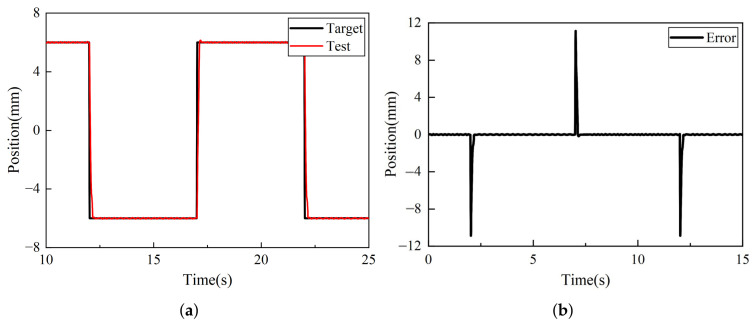

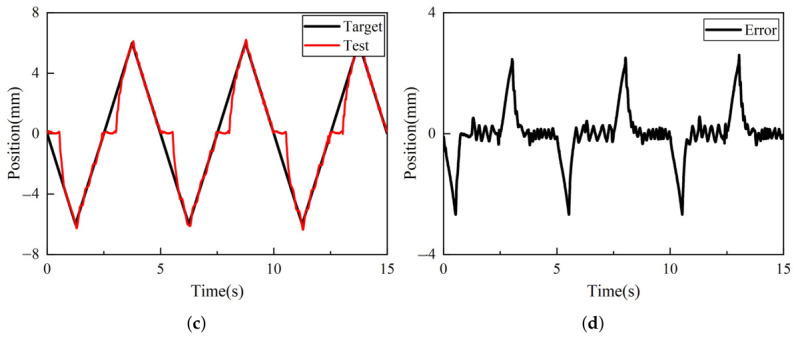
Position tracking and error responses under multi-cycle step and triangular reference inputs. (**a**) Multi-cycle step response. (**b**) Step tracking error. (**c**) Triangular reference tracking response. (**d**) Triangular tracking error.

Figure 17a depicts the tracking curve for a step signal with a frequency of 1 Hz, an amplitude of 6 mm, and a displacement of −6 mm. As evident from the diagram, the system demonstrates excellent dynamic tracking capability, with a response time of 93 ms; the valve spool responds rapidly and stabilizes at the target position. The tracking error, shown in Figure 17b, indicates a steady-state error of 0.03 mm. Figure 17c shows the tracking curve of a triangular wave signal with the same frequency, amplitude, and displacement. As shown in the figure, the system continues to demonstrate excellent dynamic tracking performance. A brief lag occurs near-zero position due to the structural dead zone inherent in the selected directional control valve. The tracking error, shown in Figure 17d, indicates a steady-state error of 0.06 mm under non-lag conditions. It should be noted that the 3 mm error observed in Figure 17d mainly occurs during spool reversal through the zero position. This phenomenon is primarily caused by the pronounced structural dead zone of the controlled valve. Because the PID controller employed in this study has limited capability in compensating for the dead-zone nonlinearity and the resulting hysteresis effect, a relatively large transient tracking error is generated in the vicinity of the zero position.

These results demonstrate that the lightweight, unconditioned circuit design proposed herein, while substantially simplifying the hardware architecture, satisfies the industrial requirements for high-precision closed-loop position control of proportional hydraulic valves.

## 7. Conclusions

This work addresses the demands for highly integrated, low-cost, and highly reliable displacement sensors in hydraulic valves within the context of Industry 4.0 and presents the design and investigation of a measurement system based on a solenoid differential self-inductance transducer. Compared with conventional LVDT/DVRT solutions employing analog synchronous demodulation, the proposed method places greater emphasis on the advantages of hardware simplification, system integration, and embedded implementation enabled by the combination of low-frequency symmetric excitation and software-based digital demodulation. The main focus of this study is to verify the feasibility and effectiveness of this lightweight scheme for hydraulic-valve position sensing and closed-loop control, which has been further validated by the application experiments in Section 6. The main conclusions are summarized as follows:

(1) The solenoid differential self-inductance transducer exhibits an excellent stroke-to-length ratio. By employing a center-tap lead configuration, the transducer achieves high sensitivity while significantly reducing its overall size, providing a physically feasible solution for the deep integration of hydraulic actuators.
(2) A lightweight signal processing scheme that eliminates the need for conventional modulation–demodulation circuitry was proposed. Both simulation and experimental results indicate that the system can interface directly with an S32K144 microprocessor, simplifying the hardware topology and demonstrating excellent linearity (2.36%) and sensitivity (155.6 mV/mm).
(3) In hydraulic valve position closed-loop control experiments, the system demonstrates superior dynamic response performance, meeting the requirements for precise spool position monitoring in intelligent hydraulic valves.

In summary, the designed displacement measurement system significantly enhances integration and cost-effectiveness without compromising measurement performance, and it shows broad application potential in high-performance proportional servo valves and intelligent hydraulic control systems. It should be noted that a more systematic quantitative comparison with conventional solutions in terms of dynamic response, noise performance, and limit of detection (LOD) remains to be carried out in future work.

## Figures and Tables

**Table 1:** The linear fit results of the voltage.

Equation	y = a + bx		
Test times	Test1	Test2	Test3
Intercept	−0.156 ± 0.013	−0.156 ± 0.011	−0.155 ± 0.012
Slope	0.259 ± 0.003	0255 ± 0.002	0.253 ± 0.002
Pearson’s r	0.998	0.998	0.998
R-square(COD)	0.999	0.999	0.999
Adj. R-square	0.998	0.999	0.999

## Data Availability

The original contributions presented in the study are included in the article; further inquiries can be directed to the corresponding author.
